# Laboratory Lathes

**Published:** 1883-11

**Authors:** 


### LABORATORY LATHES.

Those who sit at the bench while grinding, polishing, etc., will find that they can run the lathe with half the labor and with twice the force if they will have a treadle upon each side the fly-wheel, and use both feet. When but one foot is employed all the force must be applied when the crank is going through about one-fourth of its revolution, and it requires a considerable expenditure of strength to get and keep the proper momentum. But if two treadles be used the feet alternately relieve each other, and force is employed through nearly the whole revolution of the wheel.

Some years since we had such an one made by simply lengthening the shaft through the fly-wheel, putting a crank on each end and applying two treadles, and while we have had other lathes in the laboratory, they have had an almost complete vacation, as they become insufferably tiresome after the use of the double treadle.

